# Metformin and the Risk of Dementia in Type 2 Diabetes Patients

**DOI:** 10.14336/AD.2017.1202

**Published:** 2019-02-01

**Authors:** Tseng Chin-Hsiao

**Affiliations:** ^1^Department of Internal Medicine, National Taiwan University College of Medicine, Taipei, Taiwan; ^2^Division of Endocrinology and Metabolism, Department of Internal Medicine, National Taiwan University Hospital, Taipei, Taiwan; ^3^Division of Environmental Health and Occupational Medicine of the National Health Research Institutes, Zhunan, Taiwan

**Keywords:** dementia, diabetes mellitus, metformin, Taiwan

## Abstract

This retrospective cohort study investigated dementia risk associated with metformin use in type 2 diabetes patients by using the reimbursement database of the Taiwan’s National Health Insurance. The patients had new-onset diabetes during 1999-2005 and were followed up until December 31, 2011. An unmatched cohort of 147,729 ever users and 15,676 never users of metformin were identified, and a matched-pair cohort of 15,676 ever users and 15,676 never users was created by propensity score (PS). Hazard ratios were estimated by Cox regression incorporated with the inverse probability of treatment weighting using PS. Results showed that in the unmatched cohort, 713 never users and 3943 ever users developed dementia with respective incidence of 1029.20 and 570.03 per 100,000 person-years. The overall hazard ratio was 0.550 (95% confidence interval: 0.508-0.596). The hazard ratio for the first (<27.0 months), second (27.0-58.1 months) and third (>58.1 months) tertile of cumulative duration of metformin therapy was 0.975 (0.893-1.066), 0.554 (0.506-0.607) and 0.286 (0.259-0.315), respectively. Analyses in the matched cohort showed an overall hazard ratio of 0.707 (0.632-0.791) and the hazard ratio for the respective tertile was 1.279 (1.100-1.488), 0.704 (0.598-0.829) and 0.387 (0.320-0.468). In conclusion, metformin use is associated with a reduced dementia risk.

Dementia can be caused by vascular etiology or neurodegenerative disease (Alzheimer’s disease). It is a syndrome characterized by deterioration in memory and loss of daily self-care ability. It affects mainly the older people but may also happen in the younger generation. The World Health Organization (2017) has recognized the growing incidence of dementia in the world population and estimated that the number of people with dementia is currently around 47 million in the world and each year nearly 10 million new cases will add into the growing pool of patients (www.who.int/mediacentre/factsheets/fs362/en/). A call for actions and research priorities to reduce the global burden of dementia has been advocated following the First World Health Organization Ministerial Conference on Global Action Against Dementia summoned in March 2015 [1].

Elevated blood glucose may impair cerebral function and patients with diabetes have an increased risk of dementia [2]. The link between diabetes and dementia is probably multifactorial and mechanisms may involve inflammation, oxidative stress, atherosclerosis, amyloid-β deposition, brain insulin resistance with hyper-insulinemia, advanced glycation end-products (AGEs) and dysregulation of lipid metabolism [3,4].

Metformin is now considered the first-line therapy for type 2 diabetes mellitus. It reduces blood glucose level by reducing hepatic gluconeogenesis and increasing muscular glucose uptake through activation of the 5’-adenosine monophosphate-activated protein kinase (AMPK) [5]. In patients with type 2 diabetes mellitus, in addition to its glucose lowering effect, metformin has also been shown to reduce the risk of atherosclerotic events and cancers and have an anti-aging effect [6].

Studies evaluating the effect of metformin on the risk of dementia are still rare. Four population-based observational studies can be found in the literature, three from Taiwan using the administrative database of the National Health Insurance (NHI) and one from the UK using the General Practice Research Database. The first study by Hsu et al. from Taiwan showed that users of metformin only (*n*=1864, hazard ratio 0.76, 95% confidence interval 0.58-0.98) and users of metformin plus sulfonylureas (*n*=9257, hazard ratio 0.65, 95% confidence interval 0.56-0.74) had lower risk of dementia while compared to diabetes patients without taking any antidiabetic drugs (*n*=10519) [7]. The second study from Taiwan by Cheng et al. enrolled new-onset type 2 diabetes patients who had been using single oral antidiabetic drug of metformin, sulfonylureas and thiazolidinediones (TZDs), respectively [8]. When metformin users were treated as the referent group, the risk of dementia was significantly higher for users of TZDs but not for users of sulfonylureas [8]. The third study from Taiwan by Kuan et al. published recently compared 4651 metformin users and a comparable number of non-users matched on propensity score (PS) [9]. They showed a significantly higher risk in metformin users with an adjusted hazard ratio of 1.66 (95% confidence interval 1.35-2.04). The UK study by Imfeld et al. showed an increased risk of dementia associated with metformin use (odds ratio 1.71, 95% confidence interval 1.12-2.60) by using a matched case-control design including 7086 incident cases of Alzheimer’s disease and 7086 controls without dementia [10].

In a recent meta-analysis evaluating the impact of insulin sensitizers on the incidence of dementia, Ye et al. showed a statistical trend of risk reduction associated with the use of either TZDs (relative risk 0.75, 95% confidence interval 0.56-1.00, *P*=0.050) or metformin (relative risk 0.79, 95% confidence interval 0.62-1.01, *P*=0.064) [11].

Conflicting findings in the effect of metformin on cognitive function were also observed between a follow-up study conducted in Singapore and a small clinical study conducted in Australia. Ng et al. compared the cognitive function of 204 metformin users versus 161 non-users of diabetes patients recruited from the population-based Singapore Longitudinal Aging Study [12]. They showed that metformin use was associated with a lower risk of cognitive impairment (odds ratio 0.49, 95% confidence interval 0.25-0.95). In the Australian clinical study, Moore et al. showed that, among subgroup participants with diabetes (*n*=104, 35 metformin users and 91 non-users), worse cognitive performance was observed in metformin users (odds ratio 2.23, 95% confidence interval 1.05-4.75) [13].

Because metformin is widely used in a large number of diabetes patients, the conflicting findings of metformin on dementia risk and cognitive function warrant more in-depth research to clarify whether it can be beneficial or harmful. Therefore, the present study aimed at investigating the risk of dementia associated with metformin use in type 2 diabetes patients with careful consideration of potential bias and confounding commonly encountered in pharmacoepidemiological studies using existing administrative databases.

## MATERIALS AND METHODS

The National Health Insurance (NHI) was implemented in Taiwan since March 1995. It is a unique healthcare system that covers 99.6% of the Taiwan’s population and has contracts with all in-hospitals and 93% of all medical settings [14]. All records including disease diagnoses, prescribed medications and performed procedures are kept as a database, which can be used for academic research after approval by ethics review. The present study was granted an approval number 99274.

Diabetes was coded 250.XX according to the International Classification of Diseases, Ninth Revision, Clinical Modification (ICD-9-CM). Dementia was coded as abridged codes of A210 or A222, or as ICD-9-CM codes of 290.0, 290.1, 290.2, 290.4, 294.1, 331.0-331.2, or 331.7-331.9.

**Figure 1.**
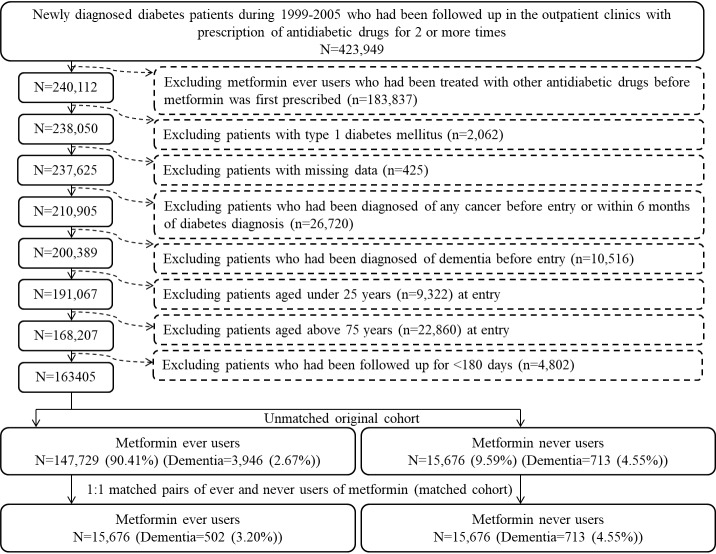
Flowchart showing the procedures followed in creating the unmatched original cohort and a cohort of 1:1 matched-pairs of metformin ever and never users from the reimbursement database of the National Health Insurance.

The database was described in more detail in previously published papers [15,16]. Figure 1 shows the procedures used to create the unmatched original cohort and the matched cohort from the database. A total of 423,949 patients diagnosed of new-onset diabetes during 1999-2005 in the outpatient clinics with prescription of antidiabetic drugs for 2 or more times were identified. Because longitudinal reimbursement data from 1996 to 2001 were available for each patient, to ensure a diagnosis of new-onset diabetes after 1999, patients with a diabetes diagnosis noted between 1996 and 1998 were not included. Ever users of metformin who had received any other antidiabetic drugs before metformin was initiated were first excluded (n=183,837). Other exclusion criteria included patients with 1) type 1 diabetes mellitus (n=2,062), 2) missing data (n=425), 3) diagnosis of any cancer before entry or within 6 months of diabetes diagnosis (n=26,720, cancer patients were excluded because of possible inclusion of distorted follow-up time due to shortened lifespan and possible misdiagnosis of dementia due to clinical presentations of malignancy), 4) diagnosis of dementia before entry (n=10,516), 5) age <25 years (n=9,322), 6) age >75 years (n=22,860) and 7) follow-up duration <180 days (n=4,802). As a result, 147,729 ever users and 15,676 never users of metformin were identified as the unmatched original cohort. The matched-pair cohort (the matched cohort) of ever and never users was created by matching the PS based on the Greedy 8 →1-digit match algorithm [17]. Logistic regression was used to create the PS from all characteristics (collected until the end of follow-up) listed in Table 1 plus the date of entry. This matching method has been used in our previous research and described in detail elsewhere [15,16].

Cumulative duration of metformin therapy in months was calculated and categorized into tertiles for dose-response analyses. Potential confounders included demographic data (age, sex, occupation and living region), major comorbidities (hypertension, dyslipidemia and obesity), diabetes-related complications (nephropathy, eye disease, stroke, ischemic heart disease and peripheral arterial disease), antidiabetic drugs (insulin, sulfonylureas meglitinide, acarbose, rosiglitazone and pioglitazone), commonly encountered comorbidities (chronic obstructive pulmonary disease, tobacco abuse, alcohol-related diagnoses, head injury and Parkinson’s disease) and commonly used medications in diabetes patients (angiotensin converting enzyme inhibitor/angiotensin receptor blocker, calcium channel blocker, statin, fibrate and aspirin). The living region and occupation were classified as detailed elsewhere [18]. In brief, the living region was classified as Taipei, Northern, Central, Southern, and Kao-Ping/Eastern. Occupation was classified as class I (civil servants, teachers, employees of governmental or private businesses, professionals and technicians), class II (people without a specific employer, self-employed people or seamen), class III (farmers or fishermen) and class IV (low-income families supported by social welfare, or veterans). The ICD-9-CM codes for the related diagnoses are provided below: hypertension (401-405), dyslipidemia (272.0-272.4), obesity (278), nephropathy (580-589), eye diseases (250.5: diabetes with ophthalmic manifestations, 362.0: diabetic retinopathy, 369: blindness and low vision, 366.41: diabetic cataract, and 365.44: glaucoma associated with systemic syndromes), stroke (430-438), ischemic heart disease (410-414), peripheral arterial disease (250.7, 785.4, 443.81 and 440-448), chronic obstructive pulmonary disease (a surrogate for smoking; 490-496), tobacco abuse (305.1, 649.0 and 989.84), alcohol-related diagnoses (291, 303, 535.3, 571.0-571.3 and 980.0), head injury (959.01) and Parkinson’s disease (332).

**Table 1:** Characteristics of metformin never users and ever users in the unmatched original cohort and in the propensity, score matched cohort.

Variable	Unmatched original cohort						Matched cohort					
	Never users		Ever users				Never users		Ever users			
	(*n*=15676)		(*n*=147729)		*P*	SD	(*n*=15676)		(*n*=15676)		*P*	SD
	n	%	n	%			n	%	n	%		
**Demographic data**												
Age* (years)	63.4±10.4		61.6±10.0		<0.01	-17.83	63.4±10.4		63.5±9.9		0.30	1.67
Sex (men)	9009	57.47	80123	54.24	<0.01	-7.18	9009	57.47	8985	57.32	0.78	-0.56
Occupation												
I	6142	39.18	58066	39.31	<0.01		6142	39.18	6084	38.81	0.92	
II	3122	19.92	34112	23.09		8.28	3122	19.92	3153	20.11		0.51
III	3301	21.06	30600	20.71		-0.65	3301	21.06	3316	21.15		0.45
IV	3111	19.85	24951	16.89		-8.58	3111	19.85	3123	19.92		0.02
Living region												
Taipei	5211	33.24	46512	31.48	<0.01		5211	33.24	5296	33.78	0.31	
Northern	1586	10.12	16577	11.22		3.71	1586	10.12	1499	9.56		-1.86
Central	2769	17.66	27115	18.35		1.88	2769	17.66	2691	17.17		-1.27
Southern	2746	17.52	25098	16.99		-1.49	2746	17.52	2780	17.73		0.65
Kao-Ping and Eastern	3364	21.46	32427	21.95		1.35	3364	21.46	3410	21.75		0.81
**Major comorbidities**												
Hypertension	12804	81.68	120731	81.72	0.89	0.27	12804	81.68	12836	81.88	0.64	0.69
Dyslipidemia	11299	72.08	122549	82.96	<0.01	28.63	11299	72.08	11290	72.02	0.91	0.26
Obesity	424	2.70	6676	4.52	<0.01	9.96	424	2.70	389	2.48	0.21	-1.29
Nephropathy	5356	34.17	40101	27.14	<0.01	-17.32	5356	34.17	5296	33.78	0.47	-1.31
Eye diseases	2942	18.77	47803	32.36	<0.01	31.61	2942	18.77	2662	16.98	<0.01	-4.94
Stroke	4996	31.87	42101	28.50	<0.01	-8.08	4996	31.87	4885	31.16	0.18	-1.49
Ischemic heart disease	7384	47.10	67018	45.37	<0.01	-3.60	7384	47.10	7348	46.87	0.68	-0.30
Peripheral arterial disease	3550	22.65	37742	25.55	<0.01	6.98	3550	22.65	3550	22.65	1.00	-0.12
**Antidiabetic drugs**												
Insulin	1282	8.18	3452	2.34	<0.01	-29.90	1282	8.18	1048	6.69	<0.01	-6.59
Sulfonylurea	11468	73.16	107896	73.04	0.75	5.13	11468	73.16	11768	75.07	<0.01	5.32
Meglitinide	1283	8.18	5767	3.90	<0.01	-19.17	1283	8.18	1259	8.03	0.62	-0.50
Acarbose	1743	11.12	8088	5.47	<0.01	-20.10	1743	11.12	1697	10.83	0.41	-1.42
Rosiglitazone	464	2.96	7388	5.00	<0.01	10.94	464	2.96	467	2.98	0.92	-0.06
Pioglitazone	387	2.47	3943	2.67	0.14	-20.10	387	2.47	398	2.54	0.69	-1.42
**Commonly encountered comorbidities**												
COPD	7675	48.96	71044	48.09	0.03	-2.12	7675	48.96	7807	49.80	0.14	1.86
Tobacco abuse	442	2.82	5943	4.02	<0.01	6.83	442	2.82	469	2.99	0.36	1.06
Alcohol-related diagnoses	1231	7.85	10490	7.10	<0.01	-4.16	1231	7.85	1087	6.93	<0.01	-3.77
Head injury	538	3.43	5524	3.74	0.05	1.44	538	3.43	523	3.34	0.64	-0.63
Parkinson’s disease	504	3.22	3349	2.27	<0.01	-6.26	504	3.22	504	3.22	1.00	0.10
**Commonly used medications in diabetes patients**												
ACEI/ARB	10854	69.24	107911	73.05	<0.01	8.81	10854	69.24	10879	69.40	0.76	-0.30
Calcium channel blocker	9771	62.33	88083	59.62	<0.01	-5.62	9771	62.33	9767	62.31	0.96	0.41
Statin	8428	53.76	97358	65.90	<0.01	26.59	8428	53.76	8300	52.95	0.15	0.06
Fibrate	5338	34.05	63817	43.20	<0.01	20.06	5338	34.05	5170	32.98	<0.05	-1.41
Aspirin	8871	56.59	90400	61.19	<0.01	9.87	8871	56.59	8797	56.12	0.40	-2.07

* Age is expressed as mean ± standard deviation. SD: standardized difference. COPD: chronic obstructive pulmonary disease, ACEI/ARB: angiotensin converting enzyme inhibitor/angiotensin receptor blocker. Refer to “Materials and Methods” for the classification of occupation

Analyses were conducted in both the unmatched original cohort and the matched cohort to examine the consistency of the findings. Student’s t test compared the difference of age between never and ever users and Chi-square test was used for other variables. Standardized difference proposed by Austin and Stuart as a test for balance diagnostics was calculated for all covariates, and a value >10% might indicate potential confounding from the variable [19].

Incidence density of dementia was calculated with regards to the use of metformin in the following subgroups: never users, ever users and the tertiles of cumulative duration. The numerator was the case number of newly diagnosed dementia identified during follow-up. The denominator was the person-years of follow-up, which ended on December 31, 2011, at the time of a new diagnosis of dementia, or on the date of death or the last reimbursement record.

Hazard ratios and their 95% confidence intervals for ever users and for each tertile of cumulative duration in referent to never users were estimated by Cox regression incorporated with the inverse probability of treatment weighting (IPTW) using the PS. As proposed by Austin, this method reduces the potential confounding from the differences in characteristics [20].

Sensitivity analyses were conducted after excluding patients who received consecutive prescriptions of metformin spanning more than 4 months and 6 months, respectively. Because the Bureau of the NHI allows at most 3 months of drug prescriptions for the patients in each outpatient visit, these analyses might have excluded most of the patients with poor adherence and did not receive regular drug refill. Incretin-based therapies were not reimbursed by the NHI until after 2009 in Taiwan. Because a recent study suggested that sitagliptin use was associated with an improvement in cognitive function [21], to avoid the potential impact of incretin-based therapies, sensitivity analysis was also conducted after excluding patients who happened to receive an incretin-based therapy during follow-up.

In consideration that more antidiabetic drugs have been introduced into clinical practice during the last two decades and the guidelines for their use have evolved over these years, the PS-weighted hazard ratios were also estimated for patients enrolled in each specific year from 1999 to 2005 in the unmatched original cohort and the matched cohort, respectively.

Analyses were conducted using SAS statistical software, version 9.3 (SAS Institute, Cary, NC). *P* < 0.05 was considered statistically significant.

**Table 2:** Incidence rates of dementia and hazard ratios by metformin exposure

Metformin use	*n*	*N*	Person-year	Incidence rate (per 100,000 person-years)	HR	95% CI	*P* value
Unmatched original cohort							
Never users	713	15676	69277.31	1029.20	1.000		
Ever users	3943	147730	691712.02	570.03	0.550	(0.508-0.596)	<0.0001
Tertiles of cumulative duration of metformin therapy (months)							
Never users	713	15676	69277.31	1029.20	1.000		
<27.0	1657	48645	168899.36	981.06	0.975	(0.893-1.066)	0.5819
27.0-58.1	1363	48872	237111.30	574.84	0.554	(0.506-0.607)	<0.0001
>58.1	923	50213	285701.36	323.06	0.286	(0.259-0.315)	<0.0001
Matched cohort							
Never users	713	15676	69277.31	1029.20	1.000		
Ever users	531	15676	72593.50	731.47	0.707	(0.632-0.791)	<0.0001
Tertiles of cumulative duration of metformin therapy (months)							
Never users	713	15676	69277.31	1029.20	1.000		
<26.6	226	5171	17707.20	1276.32	1.279	(1.100-1.488)	0.0014
26.6-57.8	180	5175	24707.24	728.53	0.704	(0.598-0.829)	<0.0001
>57.8	125	5330	30179.07	414.19	0.387	(0.320-0.468)	<0.0001

*n*: incident case number of dementia, *N*: case number followed HR: hazard ratio (weighted for propensity score), CI: confidence interval

## RESULTS

Table 1 compares the characteristics between never and ever users of metformin. In the unmatched original cohort, age and sex differed significantly. The mean age was older (63.4±10.4 vs. 61.6±10.0 years, *P*<0.01) and the proportion of men was higher (57.47% vs. 54.24%, *P*<0.01) in never users. All other variables, except hypertension, sulfonylureas, pioglitazone and head injury, also differed significantly in the original cohort. However, in the matched cohort, age and sex were similar and most variables were not different significantly (except for eye diseases, insulin, sulfonylureas, alcohol-related diagnoses and fibrate). While examining the standardized differences in the matched cohort, none had a value >10%.

The incidence of dementia and the hazard ratios by metformin exposure is shown in Table 2. The overall hazard ratios suggested a significantly lower risk of dementia associated with metformin use in either the unmatched cohort or the matched cohort. In tertile analyses, the hazard ratios suggested a reduced risk in a dose-response pattern. Patients who had used metformin for more than 2 years in the second and third tertiles consistently showed a significantly reduced risk. For the first tertile, the risk was neutral in the unmatched cohort analysis but was slightly higher with a significant *p*-value in the matched cohort.

Sensitivity analyses conducted in the unmatched cohort after excluding patients who had not received regular refill of metformin (i.e., periods between two consecutive prescriptions of metformin spanning >4 months and >6 months, respectively) or patients who happened to be treated with incretin-based therapies during follow-up did not change the conclusions of the study (Table 3).

Table 4 shows the hazard ratios for patients enrolled in each specific year from 1999 to 2005 in the unmatched cohort and the matched cohort, respectively. It is evident that the lower risk of dementia associated with metformin use was not affected by the year of enrollment.

## DISCUSSION

The findings suggested that metformin use in type 2 diabetes patients was associated with a significantly lower risk of dementia, especially when it had been used for more than 2 years (Table 2). The risk reduction showed a dose-response pattern and was consistent in sensitivity analyses (Table 3). The lower risk of dementia associated with metformin use was not affected by the year of enrollment (Table 4).

**Table 3:** Sensitivity analyses estimating hazard ratios for dementia for ever versus never users of metformin in the original cohort.

Models	*n*	*N*	HR	95% CI	*P* value
**Excluding two consecutive prescriptions of metformin spanning more than 4 months**					
Never users	713	15676	1.000		
Ever users	1046	49704	0.467	(0.425-0.514)	<0.0001
Tertiles of cumulative duration of metformin therapy (months)					
Never users	713	15676	1.000		
<27.0	392	16043	0.937	(0.826-1.064)	0.3174
27.0-58.1	325	13665	0.528	(0.463-0.603)	<0.0001
>58.1	329	19996	0.261	(0.229-0.298)	<0.0001
**Excluding two consecutive prescriptions of metformin spanning more than 6 months**					
Never users	713	15676	1.000		
Ever users	1448	65976	0.469	(0.429-0.513)	<0.0001
Tertiles of cumulative duration of metformin therapy (months)					
Never users	713	15676	1.000		
<27.0	512	19446	0.966	(0.860-1.085)	0.5606
27.0-58.1	482	19013	0.541	(0.482-0.607)	<0.0001
>58.1	454	27517	0.259	(0.230-0.292)	<0.0001
**Excluding patients treated with incretin-based therapies during follow-up**					
Never users	692	14750	1.000		
Ever users	3615	113090	0.655	(0.604-0.711)	<0.0001
Tertiles of cumulative duration of metformin therapy (months)					
Never users	692	14750	1.000		
<27.0	1580	41031	1.072	(0.979-1.173)	0.1318
27.0-58.1	1241	37153	0.650	(0.592-0.713)	<0.0001
>58.1	794	34906	0.349	(0.315-0.387)	<0.0001

*n*: incident case number of dementia, *N*: case number followed HR: hazard ratio (weighted for propensity score), CI: confidence interval

Although the mechanisms of the reduced risk of dementia associated with metformin use have not been fully investigated, some biological actions of metformin could explain such a beneficial effect. Metformin inhibits gluconeogenesis in the liver and lowers blood glucose by activating the liver kinase B1 (LKB1)/AMPK pathway through inhibiting the mitochondrial respiratory-chain complex 1 [5]. Studies suggested that activation of AMPK-dependent pathway in the brain exerts neuroprotective effects [22]. Insulin resistance with impaired insulin signaling and decreased glucose metabolism is observed in patients with dementia [23]. Metformin improves insulin resistance by increasing insulin receptor expression and improving tyrosine kinase activity [24]. A pilot randomized placebo-controlled crossover trial showed that metformin was measurable in the cerebrospinal fluid with improvement in cognitive function [25]. Increased inflammation and oxidative stress are characteristic pathophysiological changes in the brain of patients with dementia [3]. Evidence suggested that metformin may protect the cardiac and vascular system from oxidative stress and inflammation via AMPK-dependent and -independent pathways [26]. In line with such findings, animal studies supported that treatment with metformin improved cognitive function in rats with a significant reduction in inflammation and oxidative stress in the brain [27,28]. Upregulation of the mammalian target of rapamycin (mTOR) pathway has also been implicated as a major pathological process leading to Alzheimer’s disease [29]. Metformin is well known for its inhibitory effect on mTOR via activation of LKB1/AMPK [24]. Although an early laboratory study suggested that metformin increased the biogenesis of amyloid-β in neuronal tissues, which might be potentially harmful to neuronal cells, this same study showed that metformin in combination with insulin reduced amyloid-β levels [30]. More recent studies, on the contrary, suggested that metformin was neuroprotective against amyloid-β-induced mitochondrial dysfunction in human neuronal stem cells via an AMPK-dependent pathway [31] and that metformin alleviated apoptosis induced by amyloid-β via suppressing the c-Jun N-terminal protein kinases/mitogen-activated protein kinase pathway in culture hippocampal neurons [32]. AGEs can be responsible for dementia in diabetes patients with poor glycemic control [33]. Metformin can reduce the formation of AGEs through improving glycemic control and additionally it has been shown that metformin may exert a scavenging effect on AGEs [34]. Dysregulation of lipid metabolism [4] and gut microbiota dysbiosis [35] have also been implicated as potential links between diabetes and dementia. Metformin may reverse insulin resistance, improve insulin signaling and correct lipid dysmetabolism [24]. Recent studies also suggested that metformin may change the composition of gut microbiota with an increase in *Akkermansia* species leading to improvement in insulin resistance and reduction in tissue inflammation [36]. The United Kingdom Prospective Diabetes Study supported that metformin might have a cardioprotective effect resulting in reduced atherosclerotic events in obese patients with type 2 diabetes mellitus [37]. It has been well recognized that atherosclerosis plays an important role in the development of vascular dementia. Therefore, metformin may also reduce the risk of dementia through its anti-atherogenic action on the vascular system. Taken together, metformin may exert its beneficial effect on dementia via either vascular protection or neuronal protection.

**Table 4:** Hazard ratios for dementia for ever versus never users of metformin estimated for each specific year from 1999 to 2005

Year	Ever users		Never users		HR	95% CI	*P* value
	*n*	*N*	*n*	*N*			
Unmatched original cohort							
1999	793	21033	73	1335	0.562	(0.442-0.714)	<0.0001
2000	704	21309	70	1473	0.588	(0.460-0.752)	<0.0001
2001	639	22089	85	1726	0.514	(0.410-0.645)	<0.0001
2002	535	21624	100	2110	0.485	(0.392-0.600)	<0.0001
2003	502	21997	104	2411	0.511	(0.414-0.632)	<0.0001
2004	397	20430	124	2873	0.456	(0.373-0.558)	<0.0001
2005	376	19247	157	3748	0.507	(0.420-0.611)	<0.0001
Matched cohort							
1999	90	2304	73	1335	0.588	(0.432-0.801)	0.0008
2000	84	2329	70	1473	0.659	(0.480-0.906)	0.0101
2001	96	2420	85	1726	0.725	(0.541-0.971)	0.0309
2002	59	2231	100	2110	0.529	(0.383-0.730)	0.0001
2003	69	2223	104	2411	0.697	(0.514-0.945)	0.0200
2004	55	2142	124	2873	0.611	(0.445-0.840)	0.0024
2005	49	2027	157	3748	0.624	(0.453-0.861)	0.0041

*n*: incident case number of dementia, *N*: case number followed HR: hazard ratio (weighted for propensity score), CI: confidence interval

It is interesting that patients in the first tertile of short-term metformin use showed a significantly higher risk of dementia in the matched cohort analysis (Table 2). Because obesity is one of the major risk factors associated with an increased risk of dementia [38] and metformin is strongly indicated for diabetes patients with obesity [37], the increased risk in the first tertile might have been carried over from patients with obesity who were first initiated with metformin treatment.

Pharmacoepidemiological studies evaluating clinical outcomes related to medications using administrative databases may suffer from methodological limitations. These include prevalent user bias, immortal time bias and confounding by indication. Basically, these potential limitations have been carefully addressed in the present study.

The problem of prevalent user bias has been avoided by enrolling patients with newly diagnosed diabetes and new users of metformin. The potential impacts resulted from the use of other antidiabetic drugs before metformin was initiated were also avoided by including only patients who had been treated with metformin as the first antidiabetic drug in ever users (Figure 1). In consideration that the exclusion of these patients might introduce another selection bias, secondary analyses were conducted without excluding these patients. The overall hazard ratio for the unmatched cohort was 0.508 (0.471-0.549), and the hazard ratios for the respective tertiles of cumulative duration of metformin therapy were 0.894 (0.823-0.971), 0.511 (0.470-0.556) and 0.261 (0.239-0.285). For the matched cohort, the overall hazard ratio was 0.661 (0.590-0.742) and the hazard ratios for the respective tertiles were 1.210 (1.037-1.411), 0.717 (0.610-0.842) and 0.312 (0.254-0.385). Therefore, the results of the study were robust and would not be affected by the inclusion or exclusion of these patients.

Inappropriate assignment of treatment status and follow-up time may introduce immortal time bias by including the so-called immortal time (the follow-up period during which the outcome cannot happen) in the calculation of the follow-up period [39]. In the present study, it is unlikely to include ambiguous diagnosis of diabetes by enrolling only those who had been prescribed antidiabetic drugs for 2 or more times (Figure 1). The status of treatment was also less likely misclassified because the NHI is a universal healthcare system in Taiwan and all prescription information was kept for the whole period since the implementation of the NHI. Therefore, the approach used in the present study has avoided misdiagnosis of diabetes and misclassification of treatment status.

Furthermore, the exclusion of patients with a follow-up period of <180 days (Figure 1) has avoided the inappropriate assignment of follow-up time during the initial period of “immortal time”. The immortal time between diabetes diagnosis and the start of the use of antidiabetic drugs was actually not calculated in the follow-up person-years. Lévesque et al. [39] pointed out another potential source of immortal time that can be introduced during the waiting period between the prescription and dispense of medications when patients are discharged from the hospital. It is worthy to note that this would not happen in the present study because all patients were enrolled from the outpatient clinics. Even if the patients were enrolled from the hospitals, neither would this immortal time occur in Taiwan because all discharge medications can be obtained directly from the hospitals when the patients are discharged.

It is worthy to point out that immortal time might be introduced when the cumulative duration increased because the patients should have lived long enough without development of dementia up to the time of the cumulative duration. Lévesque et al. pointed out that there is a “direct relation between the immortal period and the magnitude of the bias” [39]. Therefore, the magnitude of the hazard ratios in the second and third tertiles (Table 2) should be interpreted more cautiously and the dose-response effect could not be fully clarified in the present study.

Confounding by indication could be much reduced by demonstrating the beneficial effects of metformin in both the unmatched original cohort and the PS-matched cohort (Table 2), by modeling with Cox regression incorporated with IPTW (Table 2), and by showing a lack of potential residual confounding by calculating the standardized differences and none of the covariates had a value >10% in the matched cohort (Table 1).

Small sample sizes, prevalent user bias, immortal time bias, confounding by indication, lack of dose-response analysis, and inadequate control group can be seen in earlier studies. For example, the study by Hsu et al. [7] compared the risk of dementia in subgroups of diabetes patients with the use of sulfonylureas only, metformin only and sulfonylurea plus metformin to a group of diabetes patients without ever use of any antidiabetic drugs might have included an inappropriate control group without the use of any antidiabetic drugs. Furthermore, prevalent user bias and immortal time bias were not well addressed. The study by Cheng et al. [8] included very small numbers of new-onset diabetes patients who had used solely metformin (*n*=1033), sulfonylureas (*n*=796) or TZDs (*n*=28) and compared users of sulfonylureas or TZDs to metformin users. This study has limitations of small sample sizes, lack of dose-response analysis and potential risk of immortal time bias and confounding by indication. Kuan et al. included new-onset diabetes patients identified from the cohort of the released LHID2000 database by the Bureau of NHI and defined users of metformin as any use of at least 90 days (*n*=4651) and non-users as never use of metformin (*n*=4651) during the baseline year of 2000 [9]. The LHID2000 database was formed by a cohort of 1 million insurants who joined the NHI in the year 2000 and does not include any one who was born or who joined the NHI after the year 2000. Therefore, the contamination of the use of other antidiabetic drugs for users and non-users of metformin at baseline was unavoidable during the long follow-up period to December 31, 2010. The matched case-control study by Imfeld et al. included 7086 incident cases of Alzheimer’s disease diagnosed between 1998 and 2008 and a comparable number of controls without dementia and matched on age, sex, general practice, calendar time and years of history in the UK General Practice Research Database [10]. Because of the cross-sectional nature of the case-control design, only odds ratios could be estimated, and it was not possible to completely exclude the potential risk of prevalent user bias, immortal time bias and confounding by indication in this study because these had not been well addressed. The Singaporean study by Ng et al. showing an improvement in cognitive function in users of metformin [12] and the Australian clinical study showing a significantly higher risk of dementia associated with metformin use [13] were not population-based studies. Furthermore, both enrolled very small sample sizes and evaluated cognitive function rather than dementia risk. They both certainly might suffer from the potential risk of bias and confounding commonly seen in large pharmacoepidemiological studies.

While compared to previous studies, the present study has a combined strength of including large samples of metformin users and patients with dementia, addressing most of the methodological limitations associated with pharmacoepidemiological studies and investigating the potential effect of dose-response in a follow-up design. The study has additional merits of using a nationwide database that covers >99% of the population. Therefore, the findings can be readily generalized to the whole population. The use of the medical records significantly reduced the potential biases related to self-reporting. Detection bias due to different socioeconomic status was less likely because the drug cost-sharing is low in the NHI of Taiwan and which can always be waived in patients with certain conditions like low-income household, veterans or receiving prescription refills for chronic disease.

The study limitations may include a lack of biochemical data and lack of measurement data of some confounders like anthropometric factors, smoking, alcohol drinking, lifestyle, nutritional status, dietary pattern, family history and genetic parameters (such as Apo E4 genotype). Furthermore, we did not have the data of AGEs for analyses.

In summary, the present study supports a beneficial effect of metformin on the prevention of dementia in type 2 diabetes patients. The findings give rationale for conducting clinical trials to prove such an effect. Given that metformin is safe and cheap and would not cause hypoglycemia when used as monotherapy, its usefulness for the prevention of dementia in both the diabetes patients and non-diabetes people is worthy of in-depth investigation.
